# Some Single-Machine Scheduling Problems with Learning Effects and Two Competing Agents

**DOI:** 10.1155/2014/471016

**Published:** 2014-05-25

**Authors:** Hongjie Li, Zeyuan Li, Yunqiang Yin

**Affiliations:** ^1^Mathematics Department, Zhoukou Normal University, Zhoukou 466001, China; ^2^College of Mechanical and Electrical, Harbin Institute of Technology, Harbin 150001, China; ^3^College of Sciences, East China Institute of Technology, Nanchang 330013, China; ^4^Faculty of Science, Kunming University of Science and Technology, Kunming 650093, China

## Abstract

This study considers a scheduling environment in which there are two agents and a set of jobs, each of which belongs to one of the two agents and its actual processing time is defined as a decreasing linear function of its starting time. Each of the two agents competes to process its respective jobs on a single machine and has its own scheduling objective to optimize. The objective is to assign the jobs so that the resulting schedule performs well with respect to the objectives of both agents. The objective functions addressed in this study include the maximum cost, the total weighted completion time, and the discounted total weighted completion time. We investigate three problems arising from different combinations of the objectives of the two agents. The computational complexity of the problems is discussed and solution algorithms where possible are presented.

## 1. Introduction

In traditional scheduling research, it is commonly assumed that the processing times of the jobs remain unchanged throughout the scheduling horizon. However, under certain circumstances, the job processing times may become short due to learning effects in the production environment. For example, Biskup  [1] points out that the repeated processing of similar tasks will improve workers' efficiency; that is, it takes workers shorter times to process setups, operate machines or software, or handle raw materials and components. In such an environment, a job scheduled later will consume less time than the same job when scheduled earlier. Jobs in such a setting are said to be under the “learning effect” in the literature.

Biskup [1] and Cheng and Wang [2] first introduce the idea of learning into the field of scheduling independently. Since then, a large body of literature on scheduling with learning effects has emerged. Examples of such studies are Mosheiov  [3], Mosheiov and Sidney  [4], Bachman and Janiak [5], Janiak and Rudek [6], Wang [7], and Yin et al. [8]. Biskup [9] provides a comprehensive review of research on scheduling with learning effects. For more recent studies in this line of research, the reader is referred to Jiang et al. [10, 11], Yang  [12], S.-J. Yang and D.-L. Yang  [13], Wang et al. [14], Wu et al. [15], Xu et al. [16], and Yin et al. [8].

All the above papers consider the traditional case with a single agent. In recent years scheduling researchers have increasingly considered the setting of multiple competing agents, in which multiple agents need to process their own sets of jobs, competing for the use of a common resource and each agent has its own objective to optimize. However, there is little scheduling research in the multiagent setting in which the jobs are under learning effects. Liu et al. [17] study two models with two agents and position-dependent processing times. They assume that the actual processing time of job *J*_*j*_ is *p*_*j*_ + *br* in the aging-effect model, while the actual processing time of *J*_*j*_ is *p*_*j*_ − *br* in the learning-effect model, where *r* represents the processed position of *J*_*j*_ and *b* > 0 denotes the aging or learning index. Ho et al. [18] define the actual processing time of job *J*_*j*_ as *p*_*j*_ = *a*_*j*_(1 − *kt*) if it is processed at time *t*, where *a*_*j*_ denotes the normal processing time of job *J*_*j*_ and *k* ≥ 0 represents a constant such that *k*(*t*_0_ + ∑_*j*=1_^*n*^ *a*_*j*_ − *a*_min⁡_) < 1 with *a*_min⁡_ = min⁡_*j*=1,2,…,*n*_{*a*_*j*_}. Inspired by Ho et al. [18], Yin et al. [19] consider some two-agent scheduling problems under the learning effect model proposed in Ho et al. [18], in which the objective functions for agent *A* include the maximum earliness cost, the total earliness cost, and the total weighted earliness cost, and the objective function for agent *B* is always the same, that is, maximum earliness cost, and the objective is to minimize the objective function of agent *A* while keeping the objective function of agent *B* not greater than a given level. Similar models have been further studied by Wang and Xia [20], Wang [21], and so on. For the other related two-agent works without time-dependent processing times, the reader can refer to Baker and Smith [22], Agnetis et al. [23, 24], Cheng et al. [25, 26], Ng et al. [27], Mor and Mosheiov  [28], Lee et al. [29], Leung et al. [30], Wan et al. [31], Yin et al. [19, 32], Yu et al. [33], and Zhao and Lu [34].

This study introduces a new scheduling model in which both the two-agent concept and the learning effects exist, simultaneously. We consider the following objective functions: the maximum cost, total completion time, total weighted completion time, and discounted total weighted completion time. The structural properties of optimal schedules are derived and polynomial time algorithms are developed for the problems where possible.

The remaining part of the study is organized as follows: Section 2 introduces the notation and terminology used throughout the paper. Sections 3–6 analyze the computational complexity and derive the optimal properties of the problems under study. The last section concludes the paper and suggests topics for future research.

## 2. Model Formulation

The problem investigated in this paper can be formally described as follows. Suppose that there are two agents *A* and *B*, each of whom has a set of nonpreemptive jobs. The two agents compete to process their jobs on a common machine. Agent *A* has to process the job set  *J* ^*A*^ = {*J*_1_^*A*^, *J*_2_^*A*^,…, *J*_*n*_*A*__^*A*^}, while agent *B* has to process the job set *J* ^*B*^ = {*J*_1_^*B*^, *J*_2_^*B*^,…, *J*_*n*_*B*__^*B*^}. All the jobs are available for processing at time *t*_0_, where *t*_0_ ≥ 0. Let *X* ∈ {*A*, *B*}. The jobs belonging to agent *X* are called *A*-jobs. Associated with each job *J*_*j*_^*X*^  (*j* ∈ {1,2,…, *n*_*X*_}), there are normal processing time *a*_*j*_^*X*^ and weight *w*_*j*_^*X*^. Due to the learning effect, the actual processing time *p*_*j*_ of job *J*_*j*_^*X*^ is defined as

(1) $$\begin{array}{c} p_{j}^{X}=a_{j}^{X}\left(1-kt\right),\;j=1,2,\ldots ,n_{X}, \end{array}$$

where *t* ≥ *t*_0_  denotes job's starting time and *k* ≥ 0  represents constant such that *k*(*t*_0_ + ∑_*J*_*j*_^*X*^∈*J*^*A*^∪*J*^*B*^_ *a*_*j*_^*X*^ − *a*_min⁡_) < 1, where *a*_min⁡_ = min⁡_*J*_*j*_^*X*^∈*J*^*A*^∪*J*^*B*^_{*a*_*j*_^*X*^} (see Ho et al. [18] for details).

Given a feasible schedule *S* of the *n* = *n*_*A*_ + *n*_*B*_ jobs, we use *C*_*j*_^*X*^(*S*) to denote the completion time of job *J*_*j*_^*X*^ and omit the argument *S* whenever this does not cause confusion. The makespan of agent *X* is *C*_max⁡_^*X*^ = max⁡_*j*=1,2,…,*n*_*X*__{*C*_*j*_^*X*^}. For each job *J*_*j*_^*X*^,  let *f*_*j*_^*X*^(·) be a nondecreasing function.  In this case, the maximum cost is defined as *f*_max⁡_^*X*^ = max⁡_*j*=1,2,…,*n*_*X*__{*f*_*j*_^*X*^(*C*_*j*_^*X*^)}. The objective function of agent *X* considered in this paper includes the following: *f*_max⁡_^*X*^ (maximum cost), ∑*C*_*j*_^*X*^ (total completion time), ∑*w*_*j*_^*X*^ *C*_*j*_^*X*^ (total weighted completion time), and ∑*w*_*j*_^*X*^(1 − *e* ^−*rC*_*j*_^*X*^^) (discounted total weighted completion time).

Using the three-field notation scheme *α*|*β*|*γ* introduced by Graham et al. [35], the problems considered in this paper are denoted as follows: 1 | *p*_*j*_^*X*^ = *a*_*j*_^*X*^(1 − *kt*) | *f*_max⁡_^*A*^(*C* ^*A*^) : *f*_max⁡_^*B*^(*C* ^*B*^) ≤ *U*, 1 | *p*_*j*_^*X*^ = *a*_*j*_^*X*^(1 − *kt*) | ∑*C*_*j*_^*A*^ : *f*_max⁡_^*B*^(*C* ^*B*^) ≤ *U*, 1 | *p*_*j*_^*X*^ = *a*_*j*_^*X*^(1 − *kt*) | ∑*w*_*j*_^*A*^ *C*_*j*_^*A*^ : *f*_max⁡_^*B*^(*C* ^*B*^) ≤ *U*, and 1 | *p*_*j*_^*X*^ = *a*_*j*_^*X*^(1 − *kt*) | ∑*w*_*j*_^*A*^(1 − *e* ^−*rC*_*j*_^*A*^^) : *f*_max⁡_^*B*^(*C* ^*B*^) ≤ *U*.

Note that all the objective functions involved in the considered problems are regular; that is, they are nondecreasing in the job completion times. Hence there is no benefit in keeping the machine idle.

## 3. Problem 1 | *p*_*j*_^*X*^  =  *a*_*j*_^*X*^(1 − *kt*) | *f*_max_^*A*^ : *f*_max_^*B*^ ≤ *U*

In this section we address the problem 1 | *p*_*j*_^*X*^ = *a*_*j*_^*X*^(1 − *kt*) | *f*_max⁡_^*A*^ : *f*_max⁡_^*B*^ ≤ *U* and show that it can be solved optimally in polynomial time. We first develop some structural properties of optimal schedules for the problem which will be used in developing the algorithm.

**Lemma 1 (see [19])**

For problem 1 | *p*_*j*_^*X*^ = *a*_*j*_^*X*^(1 − *kt*) | *C*_max⁡_, the makespan is equal to

(2) (t0−1k)∏j=1nX(1−kajX)+1k =(t0−1k)∏j=1nA(1−kajA)∏j=1nB(1−kajB)+1k.

In the sequel, we set *u* = (*t*_0_ − (1/*k*))∏_*j*=1_^*n*_*X*_^(1 − *ka*_*j*_^*X*^) + (1/*k*). Then the following results hold.

**Proposition 2**

For the problem 1 | *p*_*j*_^*X*^ = *a*_*j*_^*X*^(1 − *kt*) | *f*_max⁡_^*A*^ : *f*_max⁡_^*B*^ ≤ *U*, if there is a* B*-job *J*_*k*_^*B*^ such that *f*_*k*_^*B*^(*u*) ≤ *U*, then there exists an optimal schedule such that *J*_*k*_^*B*^ is scheduled last and there is no optimal schedule where an* A*-job is scheduled last.

**Proof**

Assume that *S* is an optimal schedule where the *B*-job *J*_*h*_^*B*^ is not scheduled in the last position. Let *π* denote the set of jobs scheduled prior to job *J*_*h*_^*B*^. We construct from *S* a new schedule *S* ^′  ^by moving job *J*_*h*_^*B*^ to the last position and leaving the other jobs unchanged in *S*. Then, the completion times of the jobs processed before job *J*_*h*_^*B*^ in *S*′ are the same as that in *S* since there is no change for any job preceding *J*_*h*_^*B*^ in *S*. The jobs belonging to *π* are scheduled earlier, so their completion times are smaller in *S*′ by Lemma 1. It follows that *f*_*k*_^*X*^(*C*_*k*_^*X*^(*S*′)) ≤ *f*_*k*_^*X*^(*C*_*k*_^*X*^(*S*)) for any job *J*_*k*_^*X*^ in *π*, where *X* ∈ {*A*, *B*}. By the assumption that *f*_*h*_^*B*^(*u*) ≤ *U*, job *J*_*h*_^*B*^ is feasible in *S*′,  so schedule *S*′ is feasible and optimal, as required.

For each *B*-job *J*_*j*_^*B*^, let us define a deadline *D*_*j*_^*B*^ such that *f*_*j*_^*B*^(*C*_*j*_^*B*^) ≤ *U* for *C*_*j*_^*B*^ ≤ *D*_*j*_^*B*^ and *f*_*j*_^*B*^(*C*_*j*_^*B*^) > *U* for *C*_*j*_^*B*^ > *D*_*j*_^*B*^ (if the inverse function *f*_*j*_^*B*^(·) is available, the deadlines can be evaluated in constant time; otherwise, this requires logarithmic time).

**Proposition 3**

For the problem 1 | *p*_*j*_^*X*^ = *a*_*j*_^*X*^(1 − *kt*) | *f*_max⁡_^*A*^ : *f*_max⁡_^*B*^ ≤ *U*, there exists an optimal schedule where the *B*-jobs are scheduled according to the nondecreasing order of *D*_*j*_^*B*^.

**Proof**

Assume that *S*  is an optimal schedule where the *B*-jobs are not scheduled according to the nondecreasing order of *D*_*j*_^*B*^. Let *J*_*l*_^*B*^ and *J*_*h*_^*B*^ be the first pair of jobs such that *D*_*l*_^*B*^ > *D*_*h*_^*B*^. In this schedule, job *J*_*l*_^*B*^ is processed earlier; then a set of *A*-jobs, denoted as *π*, are consecutively processed and then job *J*_*h*_^*B*^. In addition, denote by *π*′ the set of jobs processed after job *J*_*h*_^*B*^. We construct from *S* a new schedule *S*′ by extracting job *J*_*l*_^*B*^, reinserting it just after job *J*_*h*_^*B*^ and leaving the other jobs unchanged in schedule *S*. Then the completion times of the jobs processed prior to job *J*_*l*_^*B*^ in *S*′ are the same as that in *S*. By Lemma 1, the completion time of job *J*_*h*_^*B*^ in *S* equals that of job *J*_*l*_^*B*^ in *S*′; that is, *C*_*l*_^*B*^(*S*′) = *C*_*h*_^*B*^(*S*), so the completion times of the jobs belonging to *π*′ are identical in both *S* and *S*′. Since *S* is feasible, it follows that *C*_*l*_^*B*^(*S*′) = *C*_*h*_^*B*^(*S*) ≤ *D*_*h*_^*B*^ < *D*_*l*_^*B*^, so job *J*_*l*_^*B*^ is feasible in *S*′. The *π*-jobs and job *J*_*h*_^*B*^ are scheduled earlier in *S*′, implying that their actual processing times are smaller in *S*′, so their completion times are earlier in *S*′, and thus they remain feasible. Therefore, schedule *S*′ is feasible and optimal.

Thus, repeating doing this procedure for all the *B*-jobs not sequenced according to nondecreasing order of *D*_*j*_^*B*^ completes the proof.

**Proposition 4**

For the problem 1 | *p*_*j*_^*X*^ = *a*_*j*_^*X*^(1 − *kt*) | *f*_max⁡_^*A*^ : *f*_max⁡_^*B*^ ≤ *U*, if *f*_*k*_^*B*^(*u*) > *U* for any *B*-job *J*_*k*_^*B*^, then there exists an optimal schedule where the *A*-job with the smallest cost *f*_*k*_^*A*^(*u*) is processed in the last position.

**Proof**

Assume that *S* is an optimal schedule where the *A*-job with the smallest cost *J*_*h*_^*A*^, that is, *f*_*h*_^*A*^(*u*) = min⁡_*J*_*j*_^*A*^∈*J*^*A*^_{*f*_*j*_^*A*^(*u*)}, is not processed in the last position. By the hypothesis, the last job in schedule *S* is an *A*-job, say *J*_*l*_^*A*^. This means *f*_*h*_^*A*^(*u*) < *f*_*l*_^*A*^(*u*). In this schedule, job *J*_*h*_^*A*^ is scheduled earlier. Let *π* denote the set of jobs scheduled after job *J*_*h*_^*A*^ and prior to job *J*_*l*_^*A*^. We construct from *S* a new schedule *S*′ by extracting job *J*_*h*_^*A*^, reinserting it just after job *J*_*l*_^*A*^, and leaving the other jobs unchanged in schedule *S*. There is no change for any job preceding *J*_*h*_^*A*^ in *S*. We claim the following.

(1) Schedule *S*′ is feasible. First, the completion times of the jobs processed prior to job *J*_*h*_^*A*^ in *S*′ are the same as that in *S*′. Since the jobs belonging to *π* are scheduled earlier in *S*′, their actual processing times are smaller in *S*′, so their completion times are earlier in *S*′. It follows that *f*_*k*_^*X*^(*C*_*k*_^*X*^(*S*′)) ≤ *f*_*k*_^*X*^(*C*_*k*_^*X*^(*S*)) for any job *J*_*k*_^*X*^ in *π*, where *X* ∈ {*A*, *B*}, as required.
(2) Schedule *S*′ is a better schedule than *S*. By Lemma 1, the completion time of the last job *J*_*l*_^*A*^ in *S* equals that of the last job *J*_*h*_^*A*^ in *S*′; that is, *C*_*l*_^*A*^(*S*) = *C*_*h*_^*A*^(*S*′) = *u*. Thus, to prove that *S*′ is better than *S*, it suffices to show that
  (3) max⁡{flA(ClX(S′)),fhA(u)} ≤max⁡{fhA(ChX(S′)),flA(u)}.
  Since *f*_*k*_^*A*^(·) is a nondecreasing function of the completion time of job *J*_*k*_^*A*^ and *C*_*l*_^*X*^(*S*′) < *u*, we have *f*_*l*_^*A*^(*C*_*l*_^*X*^(*S*′)) ≤ *f*_*l*_^*A*^(*u*). Thus max⁡{*f*_*l*_^*A*^(*C*_*l*_^*X*^(*S*′)), *f*_*h*_^*A*^(*u*)} ≤ *f*_*l*_^*A*^(*u*), as required.

The result follows.

Summing up the above analysis, our algorithm for problem 1 | *p*_*j*_^*X*^ = *a*_*j*_^*X*^(1 − *kt*) | *f*_max⁡_^*A*^ : *f*_max⁡_^*B*^ ≤ *U* can be formally described as in Algorithm 1.

**Theorem 5**

Algorithm 1 solves problem 1 | *p*_*j*_^*X*^ = *a*_*j*_^*X*^(1 − *kt*) | *f*_max⁡_^*A*^ : *f*_max⁡_^*B*^ ≤ *U* in *O*(*n*_*A*_^2^ + *n*_*B*_log⁡*n*_*B*_) time.

**Proof**

Step 1 requires a sorting operation of the *B*-jobs, which takes *O*(*n*_*B*_log⁡*n*_*B*_) time. Step 2 takes *O*(*n*_*B*_) time since the calculation of the *f*_*j*_^*B*^(·) functions in Step 2 can be evaluated in constant time by the assumption. In Step 3 we calculate the *f*_*j*_^*A*^(·) value for all the remaining unscheduled *A*-jobs, which takes *O*(*n*_*A*_) time. Thus, after *n*_*A*_ iterations, Step 3 can be executed in *O*(*n*_*A*_^2^) time. Therefore, the overall time complexity of the algorithm is indeed *O*(*n*_*A*_^2^ + *n*_*B*_log⁡*n*_*B*_).

## 4. Problem 1 | *p*_*j*_^*X*^  =  *a*_*j*_^*X*^(1 − *kt*) | ∑*w*_*j*_^*A*^ *C*_*j*_^*A*^ : *f*_max_^*B*^ ≤ *U*

Leung et al. [30] show that problem 1 | |∑*w*_*j*_^*A*^ *C*_*j*_^*A*^ : *f*_max⁡_^*B*^ ≤ *U* is NP-hard in the strong sense. Since our problem 1 | *p*_*j*_^*X*^ = *a*_*j*_^*X*^(1 − *kt*) | ∑*w*_*j*_^*A*^ *C*_*j*_^*A*^ : *f*_max⁡_^*B*^ ≤ *U* is a generalization of the problem 1 | |∑*w*_*j*_^*A*^ *C*_*j*_^*A*^ : *f*_max⁡_^*B*^ ≤ *U*, then so is our problem. In what follows we show that the problem 1 | *p*_*j*_^*X*^ = *a*_*j*_^*X*^(1 − *kt*) | ∑*w*_*j*_^*A*^ *C*_*j*_^*A*^ : *f*_max⁡_^*B*^ ≤ *U* is polynomially solvable if the *A*-jobs have reversely agreeable weights; that is,  *a*_*i*_^*A*^ ≤ *b*_*j*_^*A*^ implies *w*_*i*_^*A*^ ≥ *w*_*j*_^*A*^ for all jobs *J*_*i*_^*A*^ and *J*_*j*_^*A*^. It is clear that Propositions 2 and 3 still hold for this problem. We modify Proposition 4 as follows.

**Proposition 6**

For the problem 1 | *p*_*j*_^*X*^ = *a*_*j*_^*X*^(1 − *kt*) | ∑*w*_*j*_^*A*^ *C*_*j*_^*A*^ : *f*_max⁡_^*B*^ ≤ *U*, if the *A*-jobs have reversely agreeable weights, then there exists an optimal schedule where the *A*-jobs are assigned according to the nondecreasing order of *a*_*j*_^*A*^/*w*_*j*_^*A*^, that is, in the weighted shortest processing time (WSPT) order.

**Proof**

Assume that *S* is an optimal schedule where *A*-jobs are not scheduled in the WSPT order. Let *J*_*l*_^*A*^ and *J*_*h*_^*A*^ be the first pair of jobs such that *a*_*l*_^*A*^/*w*_*l*_^*A*^ > *a*_*h*_^*A*^/*w*_*h*_^*A*^. Then *a*_*l*_^*A*^ ≥ *a*_*h*_^*A*^ and *w*_*l*_^*A*^ ≤ *w*_*h*_^*A*^ due to the fact that the *A*-jobs have reversely agreeable weights. Assume that, in schedule *S*, job *J*_*l*_^*A*^ starts its processing at time *T*; then a set of *B*-jobs are consecutively processed and then job *J*_*h*_^*A*^. In addition, let *π*′ denote the set of jobs processed after job *J*_*h*_^*A*^. We construct a new scheduling *S*′ from *S* by swapping jobs *J*_*l*_^*A*^ and *J*_*h*_^*A*^ and leaving the other jobs unchanged. We conclude the following.

(1) Schedule *S*′ is feasible. By Lemma 1, the completion time of job *J*_*h*_^*A*^ in *S* equals that of job *J*_*l*_^*A*^ in *S*′; that is, *C*_*l*_^*A*^(*S*′) = *C*_*h*_^*A*^(*S*), so the completion times of the jobs belonging to *π*′ are identical in both *S* and *S*′. Since *a*_*l*_^*A*^ ≥ *a*_*h*_^*A*^, we have *C*_*h*_^*A*^(*S*′) = *T* + *a*_*h*_^*A*^(1 − *kT*) ≤ *T* + *a*_*l*_^*A*^(1 − *kT*) = *C*_*l*_^*A*^(*S*). Hence the *π*-jobs are scheduled earlier in *S*′, implying that their actual processing times are smaller in *S*′, so their completion times are earlier in *S*′. Hence *f*_*k*_^*B*^(*C*_*k*_^*B*^(*S*′)) ≤ *f*_*k*_^*B*^(*C*_*k*_^*B*^(*S*)) for any job *J*_*k*_^*B*^ in *π*, as required.
(2) Schedule *S*′ is better than *S*. By the proof of (1), it is sufficient to show that
  (4) $$\begin{array}{c} w_{h}^{A}C_{h}^{A}\left(S^{\prime }\right)+w_{l}^{A}C_{l}^{A}\left(S^{\prime }\right)\leq w_{l}^{A}C_{l}^{A}\left(S\right)+w_{h}^{A}C_{h}^{A}\left(S\right). \end{array}$$
  Since *C*_*h*_^*A*^(*S*′) ≤ *C*_*l*_^*A*^(*S*) and *C*_*l*_^*A*^(*S*′) = *C*_*h*_^*A*^(*S*), we have
  (5) wlAClA(S)+whAChA(S)−(whAChA(S′)+wlAClA(S′)) ≥wlAChA(S′)+whAClA(S′)−(whAChA(S′)+wlAClA(S′)) =(wlA−whA)(ChA(S′)−ClA(S′)) ≥0,
  as required.

Thus, repeating this swapping argument for all the *A*-jobs not sequenced in the WSPT order yields the theorem.

Based on the results of Propositions 2, 3, and 6, our algorithm to solve the problem 1 | *p*_*j*_^*X*^ = *a*_*j*_^*X*^(1 − *kt*) | ∑*w*_*j*_^*A*^ *C*_*j*_^*A*^ : *f*_max⁡_^*B*^ ≤ *U* for the case where the *A*-jobs have reversely agreeable weights can be formally described as in Algorithm 2.

**Theorem 7**

The problem 1 | *p*_*j*_^*X*^ = *a*_*j*_^*X*^(1 − *kt*) | ∑*w*_*j*_ *C*_*j*_^*A*^ : *f*_max⁡_^*B*^ ≤ *U* can be solved in *O*(*n*_*A*_log⁡*n*_*A*_ + *n*_*B*_log⁡*n*_*B*_) time by applying Algorithm 2 if all *A*-jobs have reversely agreeable weights.

**Proof**

The correctness comes from the above analysis. Now we turn to the time complexity of the algorithm. Step 1 requires two sorting operations of the *A*-jobs and *B*-jobs, respectively, which take *O*(*n*_*A*_log⁡*n*_*A*_) time and *O*(*n*_*B*_log⁡*n*_*B*_) time, respectively. Both Steps 2 and 3 take *O*(2) time. Therefore, the overall time complexity of the algorithm is indeed *O*(*n*_*A*_log⁡*n*_*A*_ + *n*_*B*_log⁡*n*_*B*_).

## 5. Problem 1 | *p*_*j*_^*X*^  =  *a*_*j*_^*X*^(1 − *kt*) | ∑*w*_*j*_^*A*^(1 − *e* ^−*rC*_*j*_^*A*^^) : *f*_max_^*B*^ ≤ *U*

This section address the problem 1 | *p*_*j*_^*X*^ = *a*_*j*_^*X*^(1 − *kt*) | *w*_*j*_^*A*^(1 − *e* ^−*rC*_*j*_^*A*^^) : *f*_max⁡_^*B*^ ≤ *U*. We show that it is polynomially solvable if the *A*-jobs have reversely agreeable weights. It is clear that Propositions 2 and 3 still hold for this problem. We give Proposition 8 as follows.

**Proposition 8**

For the problem 1 | *p*_*j*_^*X*^ = *a*_*j*_^*X*^(1 − *kt*) | *w*_*j*_^*A*^(1 − *e* ^−*rC*_*j*_^*A*^^) : *f*_max⁡_^*B*^ ≤ *U*, if the *A*-jobs have reversely agreeable weights, then there exists an optimal schedule where the *A*-jobs are assigned according to the nondecreasing order of (1 − *e* ^−*ra*_*j*_^*A*^^)/*w*_*j*_^*A*^ *e* ^−*ra*_*j*_^*A*^^, that is, in the weighted discount shortest processing time (WDSPT) order.

**Proof**

We adopt the same notation as that used in the proof of Proposition 6. Assume that (1 − *e* ^−*ra*_*l*_^*A*^^)/*w*_*l*_^*A*^ *e* ^−*ra*_*l*_^*A*^^ > (1 − *e* ^−*rp*_*h*_^*A*^^)/*w*_*h*_^*A*^ *e* ^−*rp*_*h*_^*A*^^. Since *A*-jobs have reversely agreeable weights, we have *a*_*l*_^*A*^ ≥ *a*_*h*_^*A*^ and *w*_*l*_^*A*^ ≤ *w*_*h*_^*A*^. Then by the proof of Proposition 6,  we know that *C*_*h*_^*A*^(*S*′) ≤ *C*_*l*_^*A*^(*S*), *C*_*l*_^*A*^(*S*′) = *C*_*h*_^*A*^(*S*), and *C*_*k*_^*A*^(*S*′) = *C*_*j*_^*A*^(*S*) for all the other jobs *J*_*k*_^*A*^ ∈ *J*_*A*_/{*J*_*l*_^*A*^, *J*_*k*_^*A*^} and that schedule *S*′ is feasible. To show that *S*′ is better than *S*, it is sufficient to show that

(6) whA(1−e−rChA(S′))+wlA(1−e−rClA(S′)) ≤wlA(1−e−rClA(S))+whA(1−e−rChA(S)).

In fact, since *r* ∈ (0,1), *C*_*h*_^*A*^(*S*′) ≤ *C*_*l*_^*A*^(*S*), and *C*_*l*_^*A*^(*S*′) = *C*_*h*_^*A*^(*S*), we have

(7) wlA(1−e−rClA(S))+whA(1−e−rChA(S))  −(whA(1−e−rChA(S′))+wlA(1−e−rClA(S′))) =whAe−rChA(S′)+wlAe−rClA(S′)−wlAe−rClA(S)−whAe−rChA(S) ≥whAe−rChA(S′)+wlAe−rChA(S′)−wlAe−rChA(S′)−whAe−rClA(S′) =(whA−wlA)(e−rChA(S′)−e−rClA(S′)) ≥0.

Hence, *w*_*h*_^*A*^(1 − *e* ^−*rC*_*h*_^*A*^(*S*′)^) + *w*_*l*_^*A*^(1 − *e* ^−*rC*_*l*_^*A*^(*S*′)^) ≤ *w*_*l*_^*A*^(1 − *e* ^−*rC*_*l*_^*A*^(*S*)^) + *w*_*h*_^*A*^(1 − *e* ^−*rC*_*h*_^*A*^(*S*)^). Therefore, *S*′ is not worse than *S*. Thus, repeating this swapping argument for all the *A*-jobs not sequenced in the WDSPT order yields the theorem.

Based on the above analysis, our algorithm to solve the problem 1 | *p*_*j*_^*X*^ = *a*_*j*_^*X*^(1 − *kt*) | ∑*w*_*j*_^*A*^(1 − *e* ^−*rC*_*j*_^*A*^^) : *f*_max⁡_^*B*^ ≤ *U* for the case where the *A*-jobs have reversely agreeable weights can be described as in Algorithm 3.

**Theorem 9**

The problem 1 | *p*_*j*_^*X*^ = *a*_*j*_^*X*^(1 − *kt*) | ∑*w*_*j*_^*A*^(1 − *e* ^−*rC*_*j*_^*A*^^) : *f*_max⁡_^*B*^ ≤ *U* can be solved in *O*(*n*_*A*_log⁡*n*_*A*_ + *n*_*B*_log⁡*n*_*B*_) time by applying Algorithm 3 for the case that the *A*-jobs have reversely agreeable weights.

**Proof**

The proof is analogous to that of Theorem 7.

## 6. Conclusions

This paper introduced a new scheduling model on a single machine that involves two agents and learning effects simultaneously. We studied the problem of finding an optimal schedule for agent *A*, subject to the constraint that the maximum cost of agent *B* does not exceed a given value. We derived the optimal structural properties of optimal schedules and provided polynomial time algorithms for the problem 1 | *p*_*j*_^*X*^ = *a*_*j*_^*X*^(1 − *kt*) | *f*_max⁡_^*A*^ : *f*_max⁡_^*B*^ ≤ *U*. We also showed that the problems 1 | *p*_*j*_^*X*^ = *a*_*j*_^*X*^(1 − *kt*) | ∑*w*_*j*_ *C*_*j*_^*A*^ : *f*_max⁡_^*B*^ ≤ *U* and 1 | *p*_*j*_^*X*^ = *a*_*j*_^*X*^(1 − *kt*) | ∑*w*_*j*_^*A*^(1 − *e* ^−*rC*_*j*_^*A*^^) : *f*_max⁡_^*B*^ ≤ *U* can also be solved in polynomial time under certain agreeable conditions. Future research may consider the scheduling model with more than two agents.

## Figures and Tables

**Algorithm 1:**
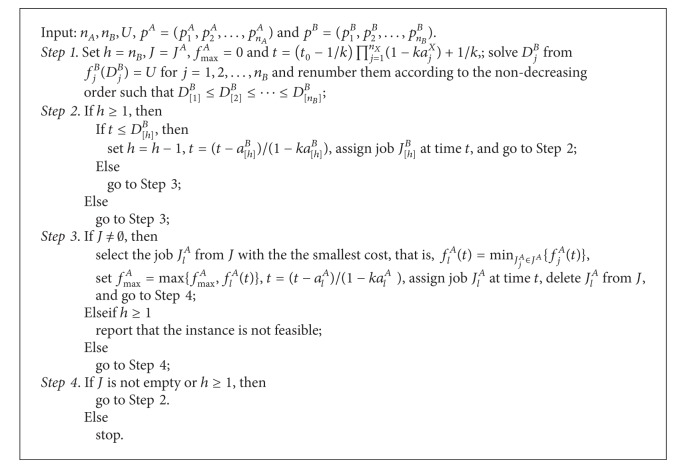


**Algorithm 2:**
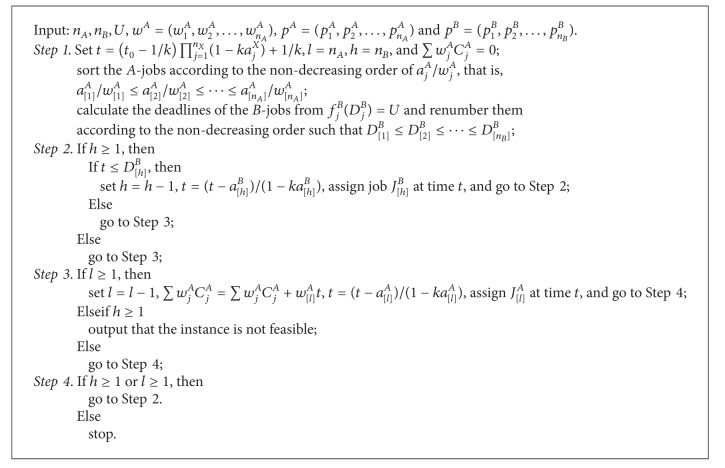


**Algorithm 3:**
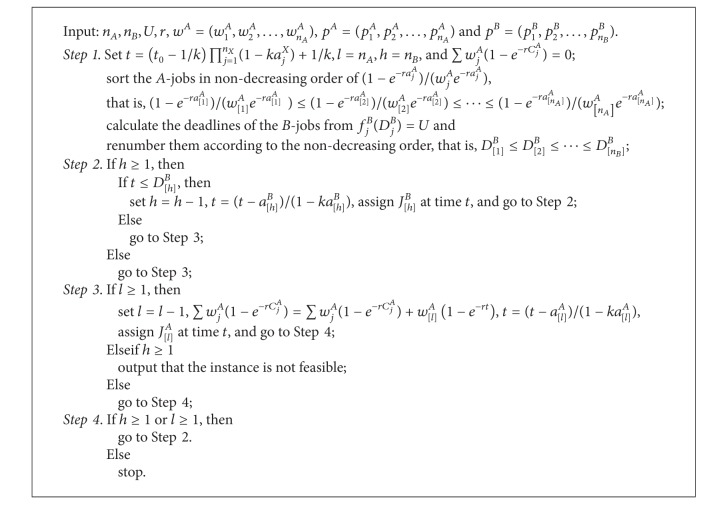


## References

[1] Biskup D (1999). Single-machine scheduling with learning considerations. *European Journal of Operational Research*.

[2] Cheng TCE, Wang G (2000). Single machine scheduling with learning effect considerations. *Annals of Operations Research*.

[3] Mosheiov G (2001). Scheduling problems with a learning effect. *European Journal of Operational Research*.

[4] Mosheiov G, Sidney JB (2003). Scheduling with general job-dependent learning curves. *European Journal of Operational Research*.

[5] Bachman A, Janiak A (2004). Scheduling jobs with position-dependentprocessing times. *Journal of the Operational Research Society*.

[6] Janiak A, Rudek R (2008). A new approach to the learning effect: beyond the learning curve restrictions. *Computers and Operations Research*.

[7] Wang J-B (2008). Single-machine scheduling with general learning functions. *Computers and Mathematics with Applications*.

[8] Yin Y, Xu D, Sun K, Li H (2009). Some scheduling problems with general position-dependent and time-dependent learning effects. *Information Sciences*.

[9] Biskup D (2008). A state-of-the-art review on scheduling with learning effects. *European Journal of Operational Research*.

[10] Jiang Z, Chen F, Kang H (2013). Single-machine scheduling problems with actual time-dependent and job-dependent learning effect. *European Journal of Operational Research*.

[11] Jiang Z, Chen F, Wu C (2012). Minimizing the maximum lateness in a single-machine scheduling problem with the normal time-dependent and job-dependent learning effect. *Applied Mathematics and Computation*.

[12] Yang S-J (2010). Single-machine scheduling problems with both start-time dependent learning and position dependent aging effects under deteriorating maintenance consideration. *Applied Mathematics and Computation*.

[13] Yang S-J, Yang D-L (2010). Minimizing the makespan on single-machine scheduling with aging effect and variable maintenance activities. *Omega*.

[14] Wang J-B, Sun L, Sun L (2010). Single machine scheduling with exponential sum-of-logarithm-processing-times based learning effect. *Applied Mathematical Modelling*.

[15] Wu C-C, Yin Y, Cheng S-R (2013). Single-machine and two-machine flowshop scheduling problems with truncated position-based learning functions. *Journal of the Operational Research Society*.

[16] Xu D, Yin Y, Li H (2010). Comments on “a note on minimizing maximum lateness in an m-machine scheduling problem with a learning effect”. *Applied Mathematics and Computation*.

[17] Liu P, Zhou X, Tang L (2010). Two-agent single-machine scheduling with position-dependent processing times. *International Journal of Advanced Manufacturing Technology*.

[18] Ho KI-J, Leung JY-T, Wei W-D (1993). Complexity of scheduling tasks with time-dependent execution times. *Information Processing Letters*.

[19] Yin Y, Cheng S-R, Wu C-C (2012). Scheduling problems with two agents and a linear non-increasing deterioration to minimize earliness penalties. *Information Sciences*.

[20] Wang J-B, Xia Z-Q (2005). Scheduling jobs under decreasing linear deterioration. *Information Processing Letters*.

[21] Wang J-B (2010). A note on single-machine scheduling with decreasing time-dependent job processing times. *Applied Mathematical Modelling*.

[22] Baker KR, Smith JC (2003). A multiple-criterion model for machine scheduling. *Journal of Scheduling*.

[23] Agnetis A, Mirchandani PB, Pacciarelli D, Pacifici A (2004). Scheduling problems with two competing agents. *Operations Research*.

[24] Agnetis A, Pacciarelli D, Pacifici A (2007). Multi-agent single machine scheduling. *Annals of Operations Research*.

[25] Cheng TCE, Ng CT, Yuan JJ (2006). Multi-agent scheduling on a single machine to minimize total weighted number of tardy jobs. *Theoretical Computer Science*.

[26] Cheng TCE, Ng CT, Yuan JJ (2008). Multi-agent scheduling on a single machine with max-form criteria. *European Journal of Operational Research*.

[27] Ng CT, Cheng TCE, Yuan JJ (2006). A note on the complexity of the problem of two-agent scheduling on a single machine. *Journal of Combinatorial Optimization*.

[28] Mor B, Mosheiov G (2010). Scheduling problems with two competing agents to minimize minmax and minsum earliness measures. *European Journal of Operational Research*.

[29] Lee K, Choi B-C, Leung JY-T, Pinedo ML (2009). Approximation algorithms for multi-agent scheduling to minimize total weighted completion time. *Information Processing Letters*.

[30] Leung JY-T, Pinedo M, Wan G (2010). Competitive two-agent scheduling and its applications. *Operations Research*.

[31] Wan G, Vakati SR, Leung JY-T, Pinedo M (2010). Scheduling two agents with controllable processing times. *European Journal of Operational Research*.

[32] Yin Y, Cheng S-R, Cheng TCE, Wu W-H, Wu C-C (2013). Two-agent single-machine scheduling with release times and deadlines. *International Journal of Shipping and Transport Logistics*.

[33] Yu X, Zhang Y, Xu D, Yin Y (2013). Single machine scheduling problem with two synergetic agents and piece-rate maintenance. *Applied Mathematical Modelling*.

[34] Zhao K, Lu X (2013). Approximation schemes for two-agent scheduling on parallel machines. *Theoretical Computer Science*.

[35] Graham RL, Lawler EL, Lenstra JK, Kan AHGR (1979). Optimization and heuristic in deterministic sequencing and scheduling: a survey. *Annals of Discrete Mathematics*.

